# Supplementary material for: ASAP-ID: Proximity Labelling With Small Tags
Source: Mol Cell Proteomics. 2026 Jun 26;25(8):101616. doi: 10.1016/j.mcpro.2026.101616 (PMC13430183; doi:10.1016/j.mcpro.2026.101616)
Supplement: Supplemental Figures [file mmc8.pdf]

Supplementary figures

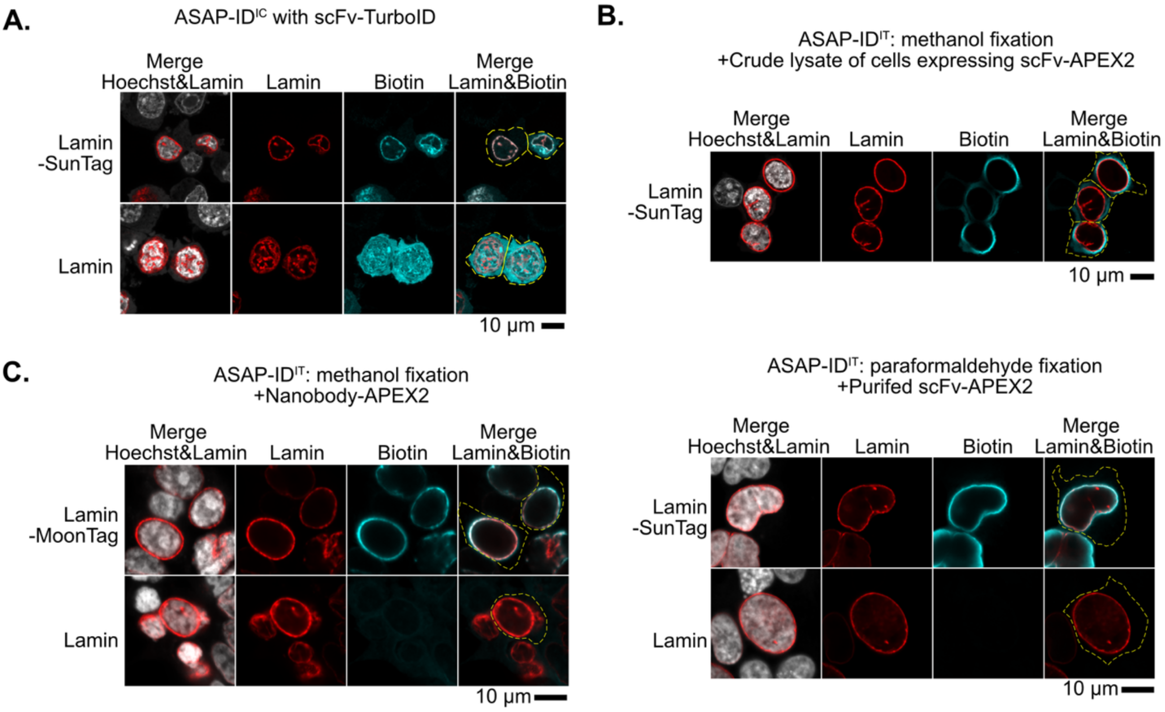

Supplementary Figure 1. ASAP-ID can label lamin proximal proteins with different approaches, biotinylation enzymes and epitope tag. A. ASAP-ID^{IC} with TurboID and SunTag. The constructs shown on the left were co-transfected with scFv fused to TurboID in HEK293T cells. Cells were then subjected to biotinylation and fixed with methanol. Representative images from two independent experiments. **B.** ASAP-ID^{IT} approaches. HEK293T cells transfected with lamin constructs (shown on left) and then fixed with methanol or paraformaldehyde, and then treated with the scFv-APEX2 fusion proteins as shown. Antibody-APEX2 was either purified using HA antibody affinity chromatography or expressed in cells, where cell lysates were added. Representative images from three independent experiments. **C.** MoonTag mediated ASAP-ID^{IT}. HEK293T cells expressed lamin constructs (shown on left) and were then fixed with methanol. Purified nanobody-APEX2 was added to fixed cells. Representative images from two independent experiments.

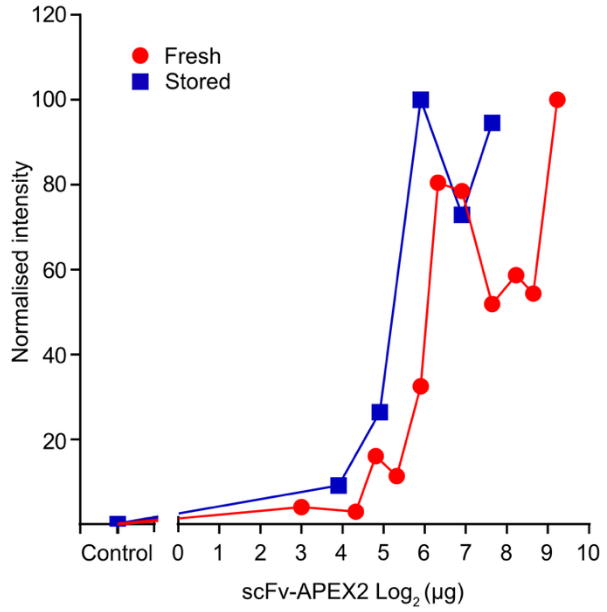

Supplementary Figure 2. Activity of purified APEX2 antibody. A. Shown are fluorescence values of biotin stain after ASAP-ID^{IT} on HEK293T cells transfected with SunTag-lamin-mCherry, and then treated with purified scFv-APEX2. Each value was derived from the average fluorescence of 12 cells. The normalised intensity was calculated by dividing the biotin immunostain fluorescence intensity by the mCherry intensity. The scFv-APEX2 was produced by immunoaffinity capture from transfected HEK293 cells. Freshly purified and snap frozen, thawed after one month storage at –80 °C, are shown.

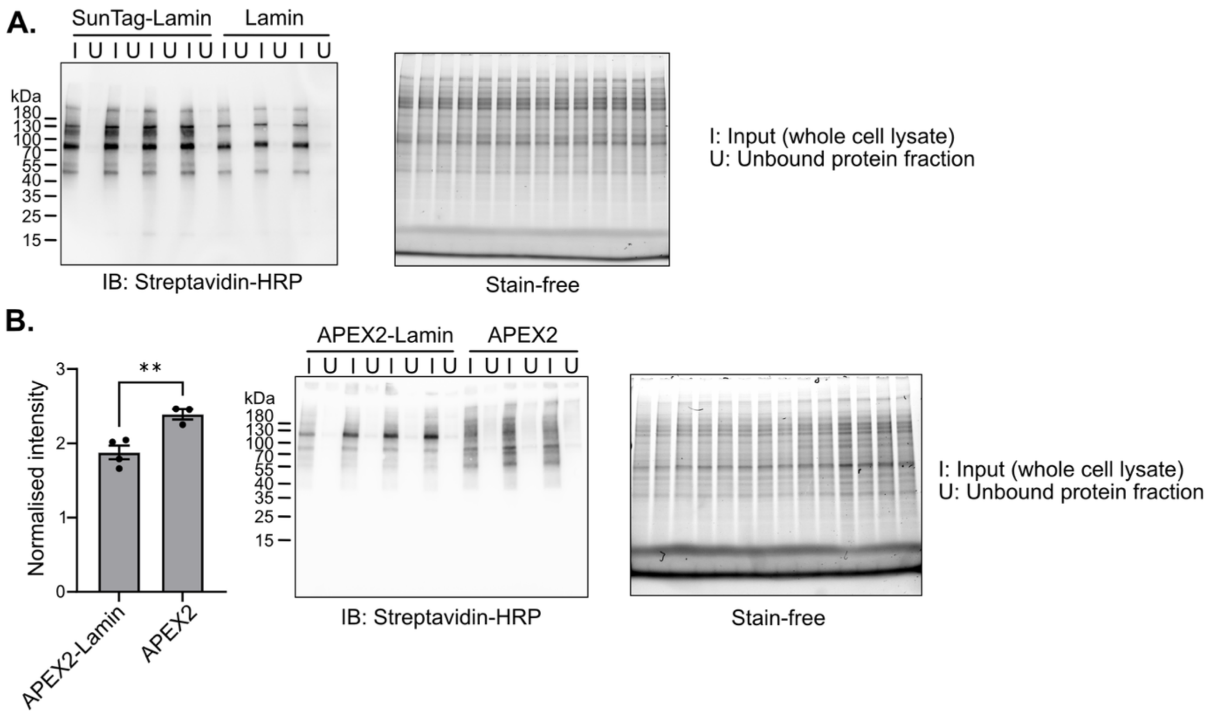

Supplementary Figure 3. Biotinylation levels in the ASAP-ID and traditional proximity labelling approach (Lamin-APEX fusion). A. Western Blot analysis of HEK293T cells expressing lamin and treated by ASAP-ID^{IT}. The biotinylated proteins were detected by the streptavidin-HRP. The unbound protein fraction was the result of a streptavidin bead pull-down of the input. **B.** Same logic as panel A, except the samples reflect APEX2-lamin fusions instead of the ASAP-ID^{IT} protocol. In this case the negative control is APEX2 alone expressed in the HEK293T cells. Graphs show densitometry data of biological replicates (dots), and the means and SEM. T-test result shown: **, P-value = 0.009.

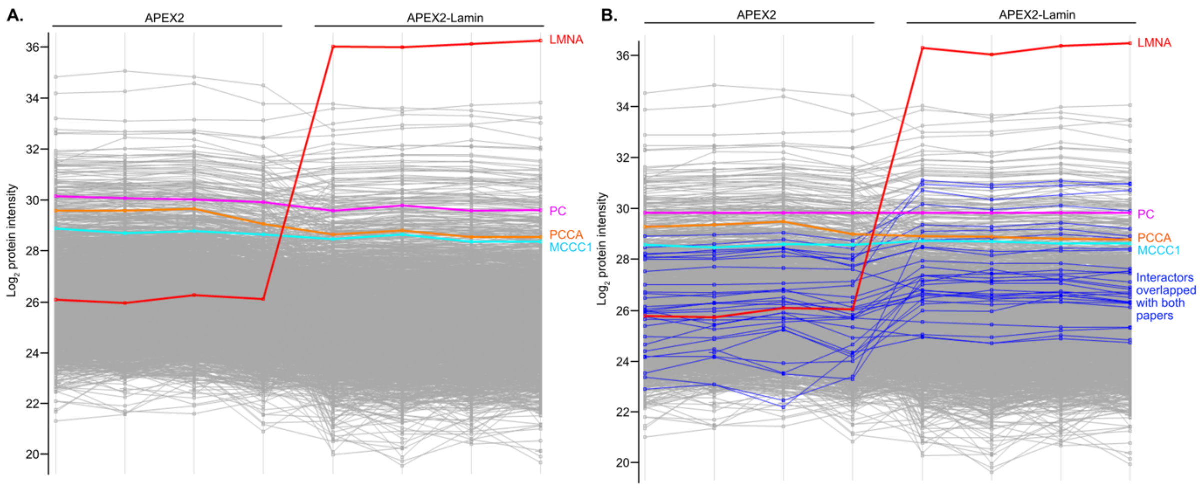

Supplementary Figure 4. Abundance profile of proteins identified in the APEX2-lamin proteomics. A. In the experiment, proximity labelling was undertaken comparing HEK293T cells expressing lamin fused to APEX with APEX alone. Data points show individual protein abundances after the filtering and imputation analysis of four replicates. Lamin and three endogenous biotinylated proteins are highlighted (PC, PCCA and MCCC1). **B.** Same as in panel A, but showing the protein profile after PC protein normalisation. Lamin interactors identified in both BioID studies (3, 17) are highlighted in blue.

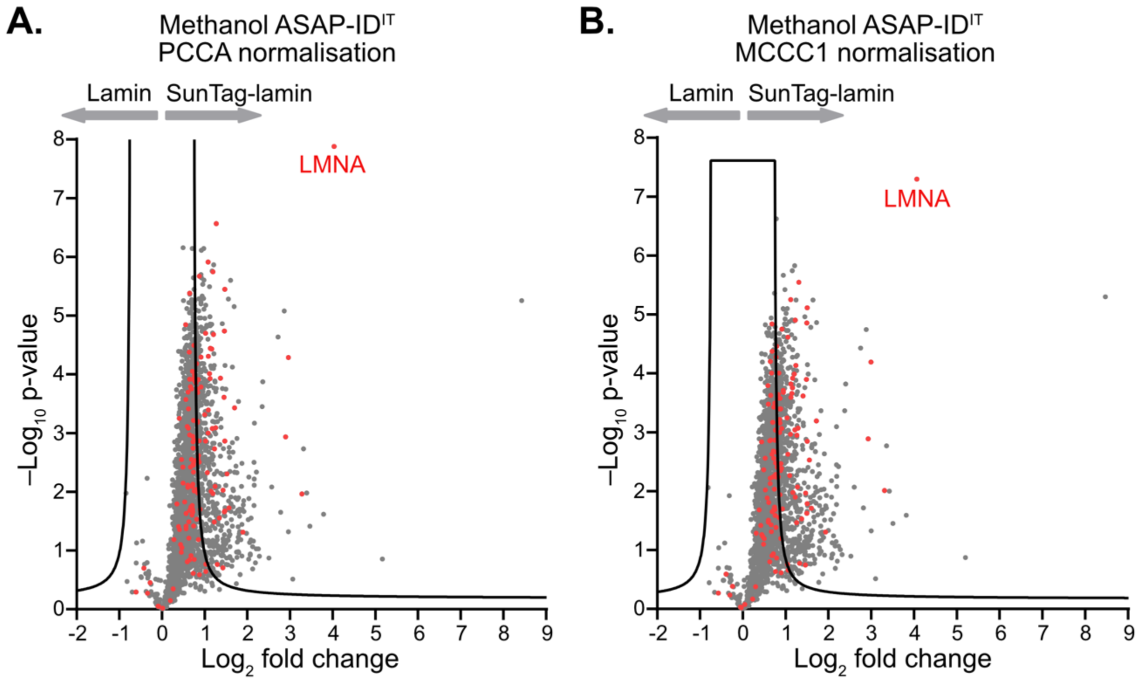

Supplementary Figure 5. Comparison of different normalisation approaches to analyse the ASAP-ID. A. ASAP-ID^{IT} on cells transfected with lamin. Data was normalised to protein PCCA abundance. Data shows lamin-SunTag/lamin changes. Red dots represent previously established lamin interactors. Threshold was set as FDR < 0.05 and S0 = 1.6. Data points are means of four biological replicates. **B**. Same logic as panel A, except the normalisation was based on protein MCCC1.

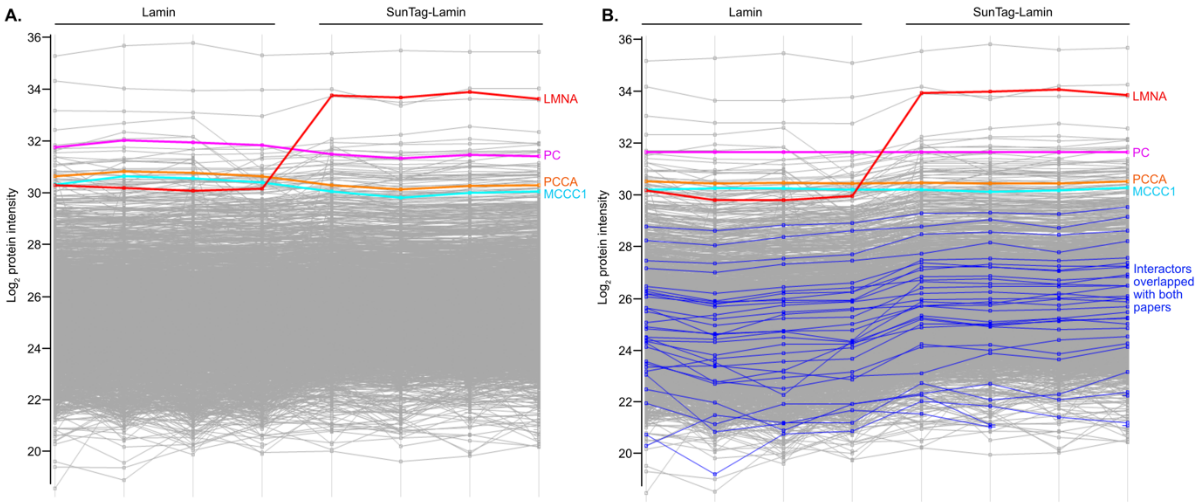

Supplementary Figure 6. Protein abundance profiles of proteins identified in ASAP-ID^{IT} using methanol fixation. A. HEK293T cells were transfected with lamin-SunTag fusion or untagged lamin. Data points show individual protein abundances after the filtering and imputation analysis of four replicates. Lamin and three endogenous biotinylated proteins are highlighted.(PC, PCCA and MCCC1). **B.** Same as in panel A, but showing the protein profile after PC protein normalisation. Lamin interactors identified in both BioID studies (3, 17) are highlighted in blue.

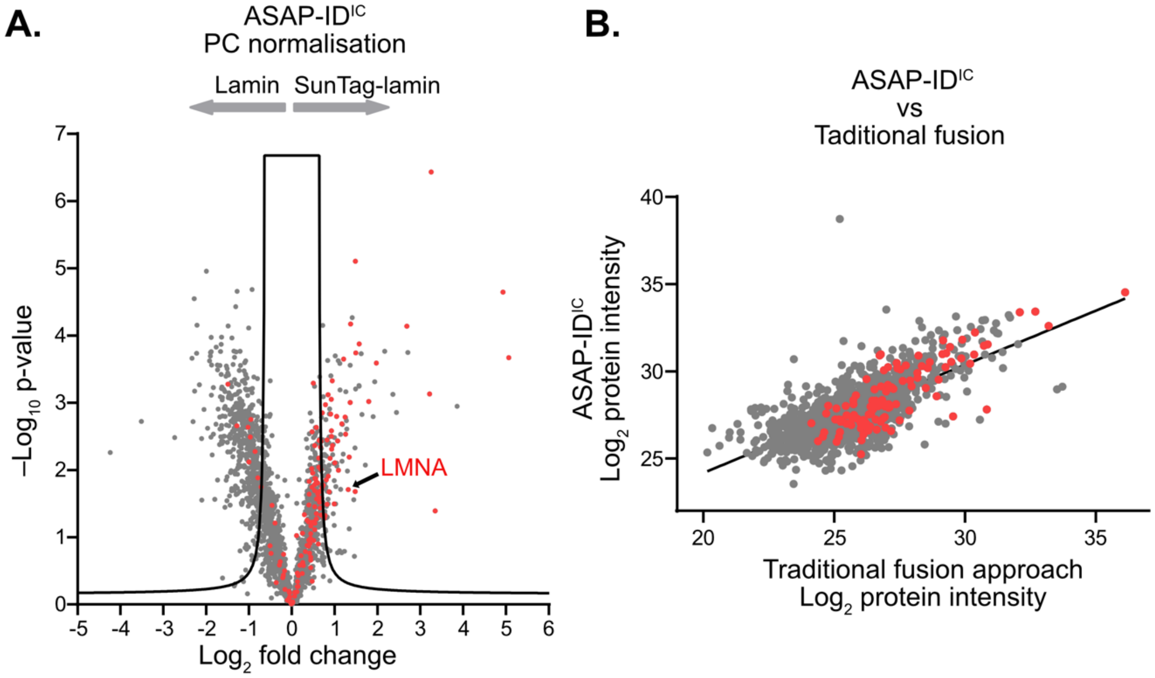

Supplementary Figure 7. Proteomics on lamin through ASAP-ID^{IC} approach. A. Volcano plot (SunTag-lamin versus untagged lamin) using PC protein normalisation in HEK293 cells. Red dots represent previously established lamin interactors. Data points are means of three biological replicates. **B.** Comparison of proteins identified by the different proximity labelling methods. The lines represent the linear regression models with R^2 values of 0.50 (traditional proximity labelling versus ASAP-ID^{IC}). Data points are means of three biological replicates.

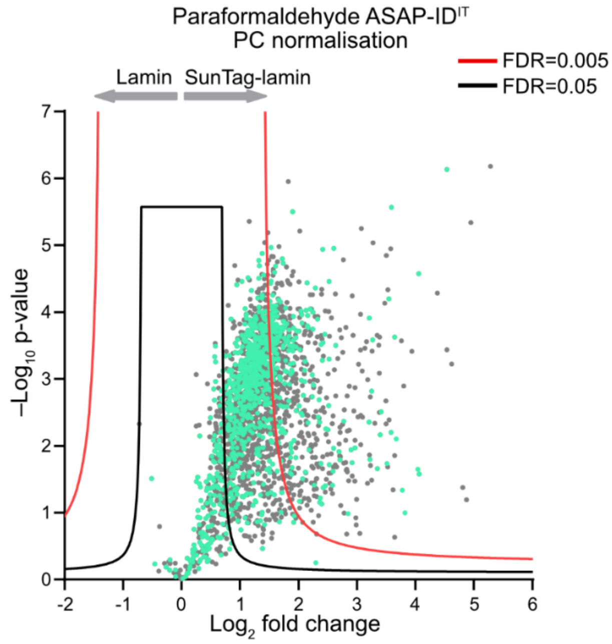

Supplementary Figure 8. Overlap of biotinylated proteins with proteins identified by ASAP-ID^{IT}. A. Volcano plot comparing SunTag-lamin with untagged lamin using paraformaldehyde ASAP-ID^{IT} in HEK293 cells. Cyan dots indicate proteins for which biotinylated peptides were identified after elution from streptavidin beads. The black line represents the threshold of FDR = 0.05, S0 = 2.5. The red line represents the threshold of FDR = 0.005, S0 = 2.5.

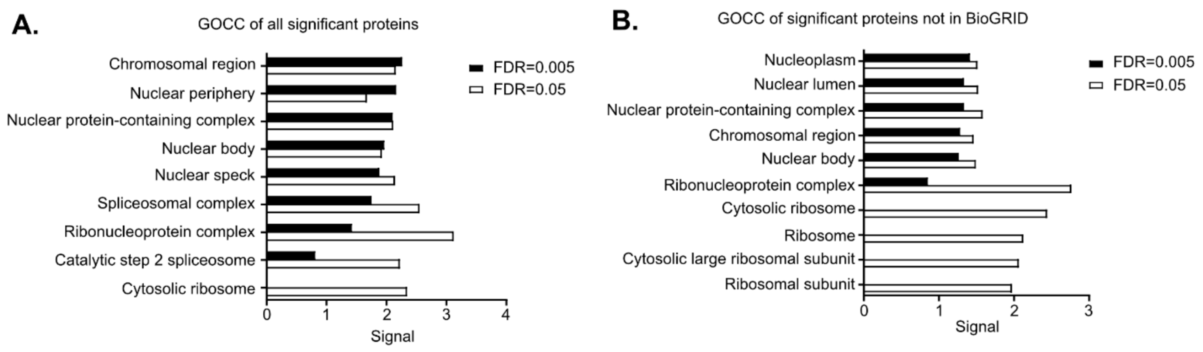

Supplementary Figure 9. Gene ontology analysis of cellular components. A. The top five cellular component terms identified under each FDR threshold in paraformaldehyde ASAP-ID^{IT}. Cellular component enrichment analysis was performed using STRING with default settings for proteins significantly enriched by ASAP-ID^{IT}. Only significantly enriched terms are shown. **B.** Same logic as panel A, except showing significantly enriched proteins that are not annotated as lamin A interactors in the BioGRID database.

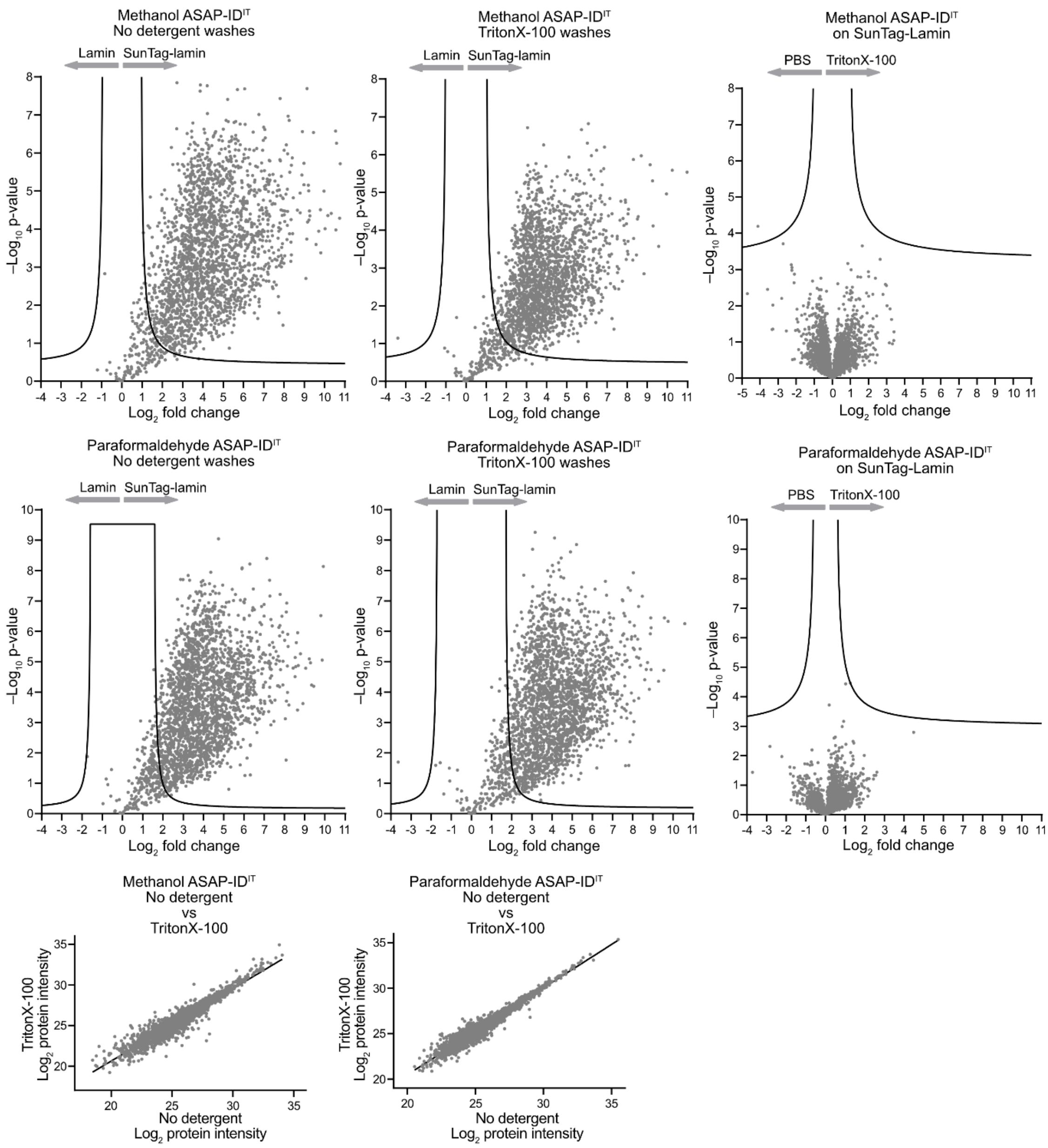

Supplementary Figure 10. Comparison of ASAP-ID^{IT} with and without detergent washing. Volcano plots comparing SunTag-lamin with untagged lamin in HEK293 cells using ASAP-ID^{IT} with or without a 0.1% Triton X-100 wash step before biotinylation. Methanol and paraformaldehyde ASAP-ID^{IT} conditions are shown. Protein intensities were normalised to PC protein levels. For comparisons between SunTag-lamin and untagged lamin in methanol ASAP-ID^{IT}, with or without detergent washing, the significance threshold was set to FDR = 0.01 and S0 = 1. For the corresponding paraformaldehyde ASAP-ID^{IT} comparisons, the threshold was set to FDR = 0.01 and S0 = 4. For volcano plots comparing Triton X-100 wash and PBS wash conditions in SunTag-lamin samples, the default threshold of FDR = 0.05 and S0 = 0.1 was used, as proteins were expected to be detected in both conditions. The bottom panel showed a comparison of mean protein $\log_2$ intensities between the no-detergent wash and Triton X-100 wash conditions. Lines represent linear regression models, with R^2 values of 0.90 for methanol ASAP-ID^{IT} and 0.94 for paraformaldehyde ASAP-ID^{IT}.

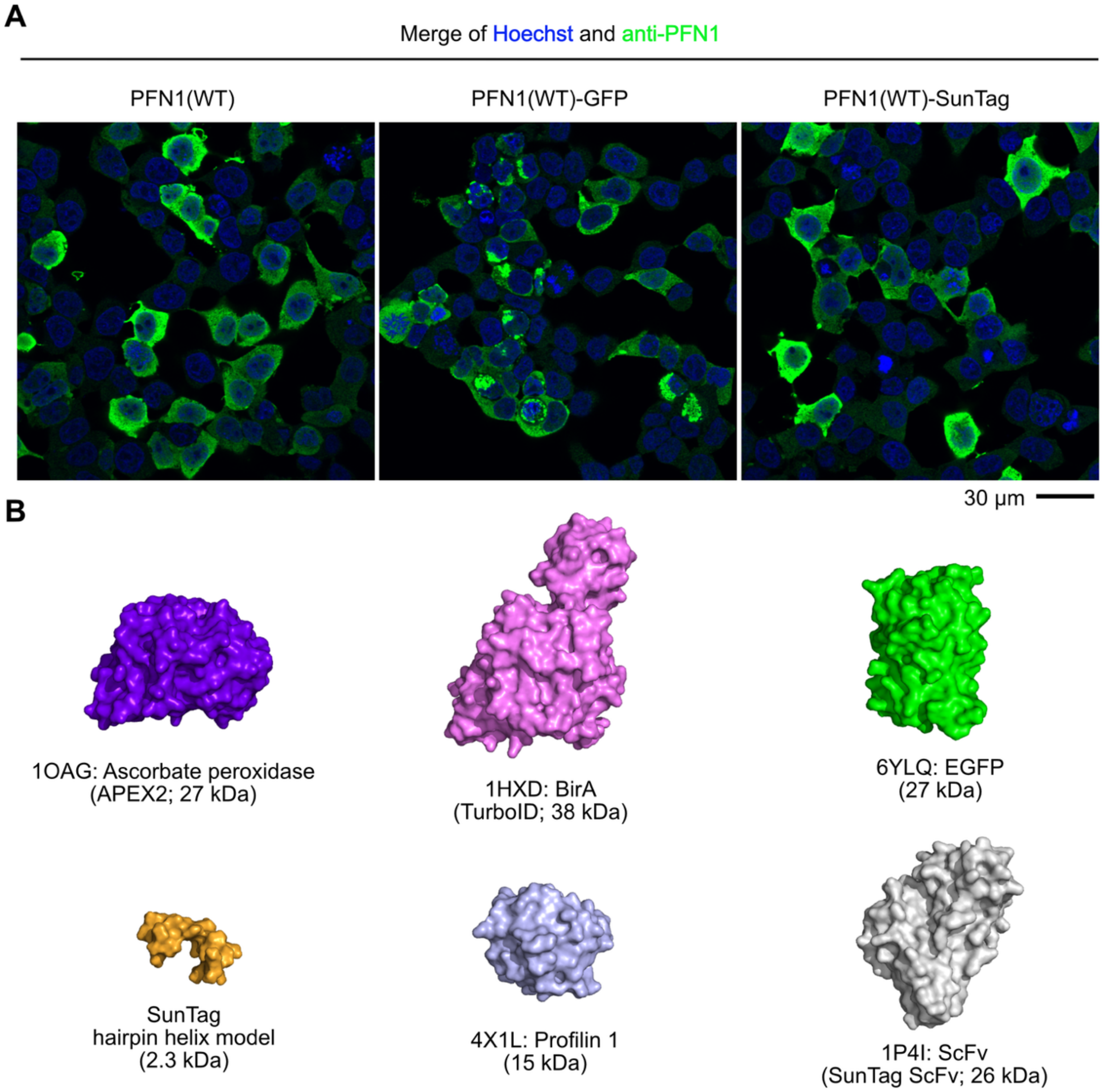

Supplementary Figure 11. Comparison of the expression of different tagged wild-type PFN1. A. Immunostaining of the GFP-tagged, SunTag-tagged tagged and nontagged wild-type PFN1. HEK293T cells were transfected with different constructs of tagged PFN1, cells were then fixed with paraformaldehyde and stained with anti-PFN1 antibody. Representative images from one experiment. **B.** Renders of proteins for consideration of scale. Protein models are shown with relevant PDB entries, and are shown to scale. Rendering was performed by PyMol (https://www.pymol.org/).

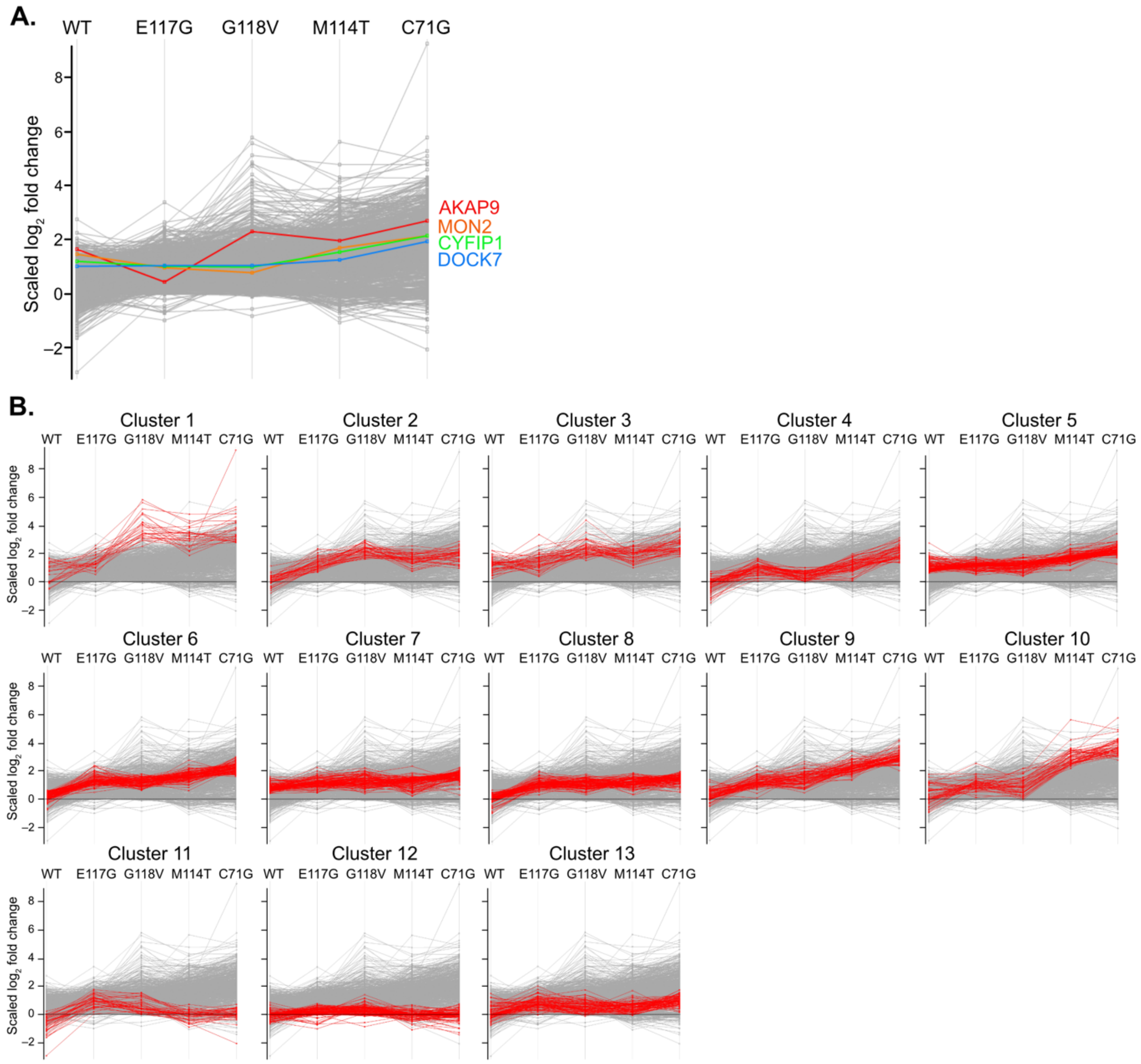

Supplementary Figure 12. K-Means cluster profiles of proteins identified in the PFN1 ASAP-ID^{IT} proteomics. A. The abundance profiles of selected known PFN1-interacting proteins. Data points indicate different protein abundances after smoothing of replicates using PERCEPT to highlight the important changes (refer to methods). **B.** The complete list of 13 clusters (in red) generated by the k-means clustering analysis.

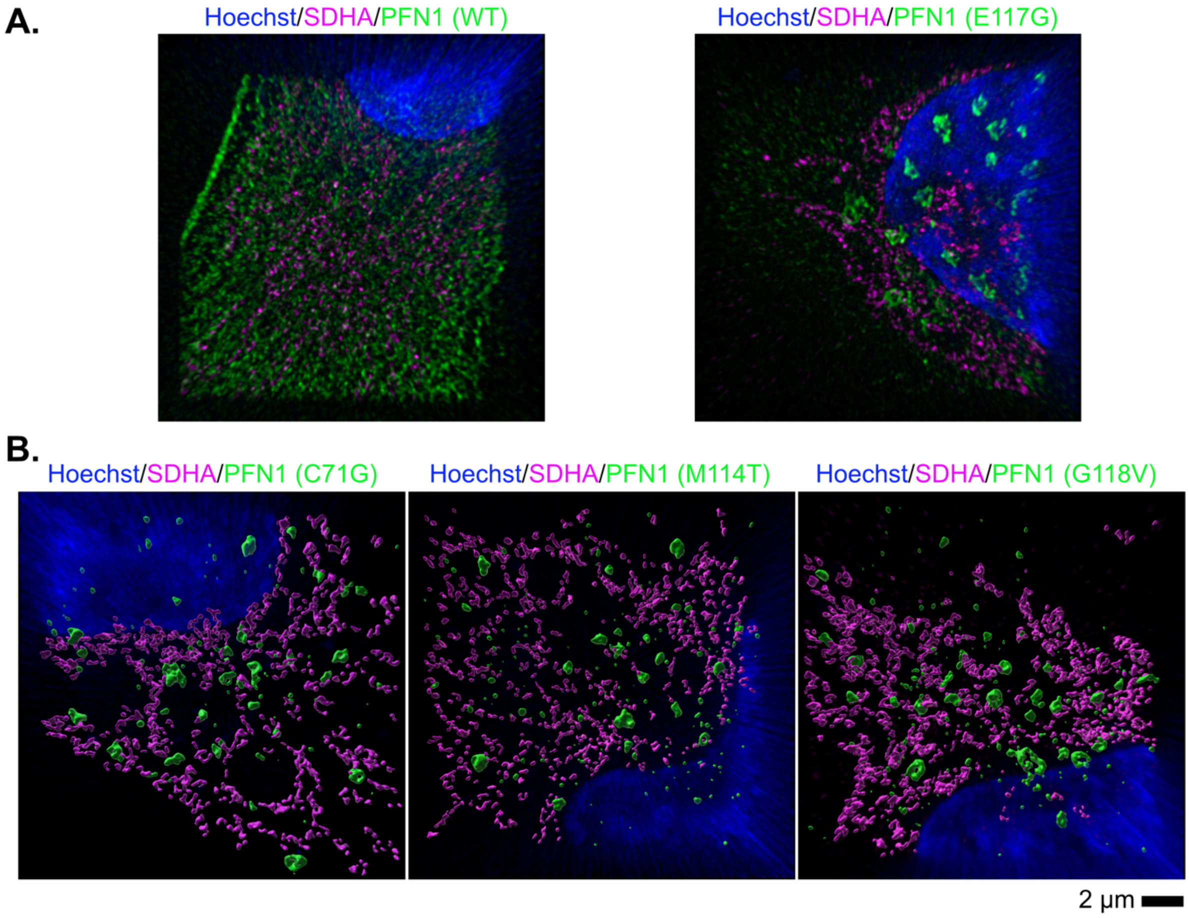

Supplementary Figure 13. Super-resolution microscope images of PFN1 aggregates and SDHA. A. Images of WT and E117G PFN1 with SDHA detected by immunostaining and SIM super-resolution microscopy. HeLa cells were transfected with HA-tagged PFN1 mutants. Cells were fixed with methanol and stained with anti-SDHA and anti-HA antibodies to detect the SDHA and PFN1 in cells. B. Same as in panel A, except the surfaces of PFN1 aggregates and SDHA protein were reconstructed using Imaris image analysis software.
